# ﻿The genus *Erhaia* (Gastropoda, Truncatelloidea, Amnicolidae), with a new species from Bhutan

**DOI:** 10.3897/zookeys.1085.77900

**Published:** 2022-02-02

**Authors:** Edmund Gittenberger, Choki Gyeltshen, Björn Stelbrink

**Affiliations:** 1 Naturalis Biodiversity Center, P.O. Box 9517, NL-2300 RA Leiden, Netherlands Naturalis Biodiversity Center Leiden Netherlands; 2 GiMaRIS, Rijksstraatweg 75, NL-2171AK Sassenheim, Netherlands GiMaRIS Sassenheim Netherlands; 3 National Biodiversity Centre, Serbithang, Thimphu, Bhutan National Biodiversity Centre Thimphu Bhutan; 4 Department of Animal Ecology & Systematics, Justus Liebig University Giessen, Heinrich-Buff-Ring 26 (IFZ), D-35392 Giessen, Germany Justus Liebig University Giessen Giessen Germany

**Keywords:** 16S, Bhutan, China, COI, *Erhaia*, India, Nepal, taxonomy

## Abstract

The distribution of the five *Erhaia* (Gastropoda, Truncatelloidea, Amnicolidae) species that are diagnosed by both morphological and molecular data is combined with several records of less completely diagnosed nominal *Erhaia* species. The resulting distribution pattern is summarized in a map and is discussed herein. *Erhaianorbui***sp. nov.** is described from Bhutan on the basis of shell morphology and two mitochondrial DNA barcoding markers. A molecular phylogeny is presented for the five *Erhaia* species for which molecular data are available, three of which form a separate clade and are from Bhutan.

## ﻿Introduction

The genus *Erhaia* Davis & Kuo, 1985 (Gastropoda, Truncatelloidea, Amnicolidae), as it is accepted in the literature at present (Gittenberger et al. 2020 and literature therein), is distributed over an area covering nearly 3.500 km from west to east, from northern India and Nepal to eastern China. Comparable to its European counterpart *Bythinella* Moquin-Tandon, 1856 (Gastropoda, Truncatelloidea, Bythinellidae), which is known from an even larger area measuring nearly 4.000 km from west to east, from Spain to western Russia and Ukraine (Boeters 1998; Vinarski and Kantor 2016), it exemplifies a radiation, in which some species occur syntopically, that may have evolved in a non-adaptive fashion (see Gittenberger 1991; Wilke et al. 2010). However, the lack of data does not allow for a more fundamental discussion here.

Both *Erhaia* and *Bythinella* species occur in the clear waters of springs and brooklets. Despite their large ranges, suggesting relatively easy dispersal mechanisms, i.e., low barriers to gene flow, both genera show a high degree of allopatric speciation. This is illustrated by the occurrences in Bhutan, where four species, including the one described as new below, are known only from a single locality. At one locality, two of these species occur syntopically. Three *Erhaia* species are reported from the Latipur and Kavre districts in the province of Bagmati in Nepal (Nesemann et al. 2007); two of them are known from one locality only, where they occur together with the third species, which has been reported from four additional localities, thus from six in total. A taxonomic revision is needed to clarify whether the Chinese species have similar small ranges and syntopic occurrences.

The shells of species in these two genera are more or less slender ovoid and less than 5 mm high. They show a conspicuous transition in height-width ratio from protoconch to teleoconch. The protoconch shell is valvatiform, as for example in fully grown freshwater snails of the species *Valvatacristata* O.F. Müller, 1774 (Gastropoda, Valvatoidea, Valvatidae) (Glöer 2019: 196, fig. 244), whereas the teleoconch is not, therefore the shells have an obliquely flattened apical part. The adaptive significance of this, if any, is unknown. Fully grown valvatiform or planispiral shells occur in several species of minute spring snails (e.g. Hershler and Longley 1986; Beran et al. 2014). Apart from their general shape, shells of *Erhaia* vary more than those of *Bythinella* and may have character states that do not occur in that genus, viz. a spiral microsculpture and one or more lamellae inside the shell.

## ﻿Material and methods

**Figure 1.**
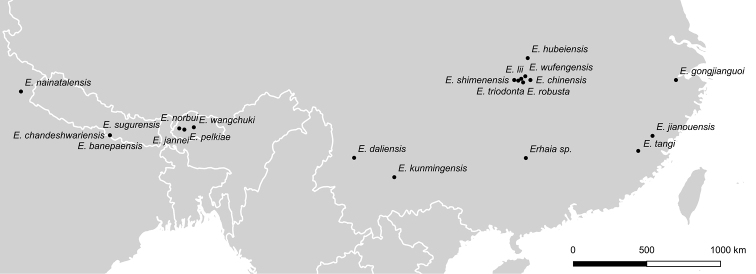
Distribution of *Erhaia* species across Asia.

Using the literature, we compiled distributional records for 22 nominal species (and a single undetermined individual from China) that are currently classified more or less convincingly in *Erhaia* (Fig. 1). Many of these taxa were originally classified in *Bythinella* or *Pseudobythinella* Liu & Zhang, 1979 (Gastropoda, Truncatelloidea, Bythinellidae) (not *Pseudobythinella* Melville, 1956). A taxonomic revision, which is beyond the scope of the present study and is also currently not possible given the lack of molecular data for many species, may indicate that some nominal taxa are synonyms. The original descriptions of all taxa mentioned here are included in the References. Following Davis et al. (1985) and Davis and Rao (1997), we excluded so-called *Bythinella* taxa described from Japan. Coordinates for each species were obtained from the source publication or were estimated based on the locality information provided therein (Table 1). Distribution maps were generated using QGIS 3.10.5 (QGIS Development Team 2000).

**Table 1.** Distribution of *Erhaia* species with coordinates (sorted from west to east) either provided by the source publication or estimated based on the locality information therein.

Species	Coordinates
**Bhutan**	
*E.norbui* spec. nov.	27°22'33.0"N, 89°17'15.0"E
*E.jannei* Gittenberger & Stelbrink in Gittenberger et al., 2020	27°18'43.0"N, 89°36'10.0"E
*E.pelkiae* Gittenberger & Gyeltshen in Gittenberger et al., 2020	27°18'43.0"N, 89°36'10.0"E
*E.wangchuki* Gittenberger, Sherub & Stelbrink, 2017	27°26'17.6"N, 90°11'18.9"E
**Elsewhere**	
*E.nainatalensis* Davis & Rao, 1997	29°23'00.0"N, 79°30'00.0"E
*E.banepaensis* Nesemann & S. Sharma in Nesemann et al., 2007	27°00'00.0"N, 85°00'00.0"E
*E.chandeshwariensis* Nesemann & S. Sharma in Nesemann et al., 2007	27°00'00.0"N, 85°00'00.0"E
*E.sugurensis* Nesemann, Shah & Tachamo in Nesemann et al., 2007	27°00'00.0"N, 85°00'00.0"E
*E.daliensis* Davis & Kuo in Davis et al., 1985	25°45'00.0"N, 100°06'00.0"E
*E.kunmingensis* Davis & Kuo in Davis et al., 1985	24°40'00.0"N, 102°35'00.0"E
*E.lii* (Kang, 1985) [also in Kang, 1986]	30°00'00.0"N, 110°00'00.0"E
*E.shimenensis* (Liu, Zhang & Chen, 1982)	30°00'00.0"N, 110°00'00.0"E
*E.triodonta* (Liu, Wang & Zhang, 1991)	29°58'00.0"N, 110°15'00.0"E
*E.wantanensis* (Kang, 1983a)	30°04'00.0"N, 110°26'00.0"E
*E.robusta* (Kang, 1986)	29°52'18.8"N, 110°32'54.5"E
*E.wufungensis* (Kang, 1983a)	30°12'00.0"N, 110°41'00.0"E
*Erhaia* sp. [Liu et al. 2014: Table 5]	25°44'16.0"N, 110°43'07.0"E
*E.hubeiensis* (Liu, Zhang & Wang, 1983)	31°10'00.0"N, 110°50'00.0"E
*E.chinensis* (Liu & Zhang, 1979)	30°00'00.0"N, 111°00'00.0"E
*E.liui* (Kang, 1985)	30°00'00.0"N, 111°00'00.0"E
*E.tangi* (Cheng, Wu, Li & Lin, 2007)	26°08'00.0"N, 117°40'00.0"E
*E.jianouensis* (Liu & Zhang, 1979)	26°58'00.0"N, 118°33'00.0"E
*E.gongjianguoi* (Kang, 1983b)	30°00'00.0"N, 120°00'00.0"E

**Figure 2.**
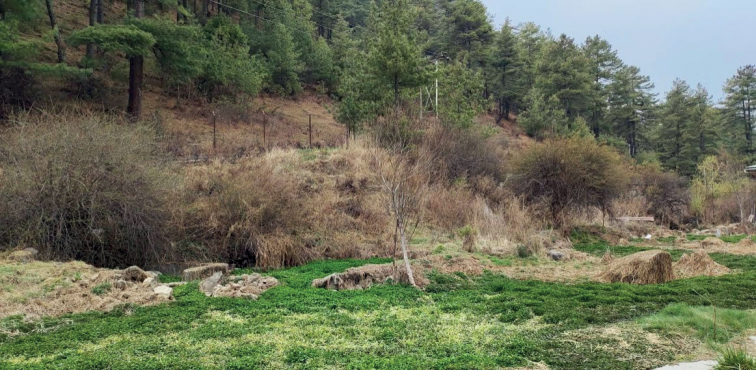
Habitat of *E.norbui* sp. nov. at the type locality. Photo by Mr. Sangay Norbu.

**Figures 3–5.**
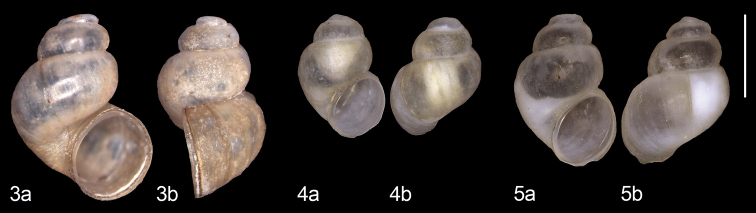
*Erhaianorbui* sp. nov. from the type locality, district Haa, Uesu, Naychu, ca. 2700 m a.s.l. **3** holotype, NBCB 1239 (H = 2.3 mm) and paratypes used for DNA analyses (**4** UGSB 25956, H = 1.5 mm **5** UGSB 25957, H = 1.8 mm). Scale bar: 1 mm.

In a spring area in Bhutan (Fig. 2), several specimens of a minute snail species were discovered and collected by Sangay Norbu. Based on this material, *Erhaianorbui* sp. nov. is described here. Photographs of the holotype (Fig. 3) were made using a Wild MS-26 binocular camera set-up. Shells of two paratypes (Figs 4, 5), which were used for a molecular analysis and thus could not be saved, were photographed with a Keyence VHX-2000 digital microscope system (Keyence Corp., Itasca, IL, USA). Additional paratypes were kept as dry shells.

The DNA lab work and phylogenetic analyses were identical to those described in Gittenberger et al. (2020). For the phylogenetic analyses, a reduced dataset including both mitochondrial markers, COI and 16S rRNA, was used. Uncorrected genetic p-distances for COI and 16S rRNA between the species from Bhutan were calculated using MEGA X 10.1.7 (Kumar et al. 2018).

The following abbreviations are used: B = shell breadth; H = shell height; NBCB = National Biodiversity Centre, Serbithang, Thimphu, Bhutan; RMNH = National Biodiversity Center Naturalis, Leiden, The Netherlands.

## ﻿Systematics

### ﻿Superfamily Truncatelloidea Gray, 1840

**Family Amnicolidae Tryon, 1863**

#### ﻿Genus Erhaia

Taxon classificationAnimaliaLittorinimorphaAmnicolidae

Davis & Kuo in Davis et al., 1985

##### Type species by original designation.

*Erhaiadaliensis* Davis & Kuo in Davis et al., 1985.

##### Synonym.

*Pseudobythinella* Liu & Zhang, 1979. Not Melville, 1956. Type species by original designation: *Pseudobythinellajianouensis* Liu & Zhang, 1979.

##### Description.

Shell ovoid to elongate ovoid or conical, smooth or with spiral microsculpture on the proto- and/or teleoconch. Apex conspicuously and more or less obliquely flattened. Aperture varying from ovoid-elliptical to circular; its palatal side curved and gradually passing into the basal side. Peristome continuous, attached at the parietal side or more or less protruding. Umbilicus minute or closed. Parietal part of the aperture smooth or with a lamella; columella smooth or with 2 spiral lamellae.

##### Notes.

Molecular data, which are available for only a limited number of the amnicolid species, are inconclusive regarding the status of *Erhaia* versus *Akiyoshia* Kuroda & Habe, 1954 Gastropoda, Truncatelloidea, Amnicolidae) (see also the more comprehensive phylogenetic reconstruction in Gittenberger et al. 2020). No DNA data are known for the type species of these nominal taxa, i.e., *E.daliensis* Davis & Kuo, 1985 and *A.uenoi* Kuroda & Habe, 1954. Furthermore, the species that are generally called *Erhaiajianouensis* (Liu & Zhang, 1979) and *Akiyoshiakobayashii* Kuroda & Habe, 1958 are sister species (Fig. 7) that should be congeneric by definition. At present, their ranges in China and Japan, respectively, have been decisive for the generic classification. Pending additional data that can help solve this problem convincingly, we opted to still use the current, contradictory nomenclature (see also notes under *E.norbui* sp. nov.).

##### Distribution.

**Figure 6.**
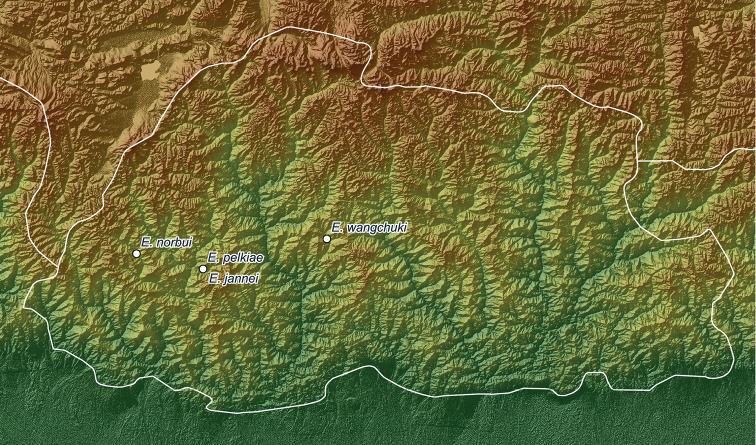
Distribution of *Erhaia* species described for Bhutan. Note that *E.jannei* and *E.pelkiae* were found to occur syntopically.

The genus *Erhaia* was initially reported from a wide range in southern China, where it has been recorded with various species from the provinces of Yunnan, Sichuan, Guangxi, Hubei, Hunan, and Fujian (Davis et al. 1985; Davis and Kang 1995; Davis and Rao 1997; Wilke et al. 2000, 2001; Liu et al. 2014). Regarding its occurrence in Japan, see foregoing notes. One species was described from northern India (Davis and Rao 1997), three additional species were described from Nepal (Nesemann et al. 2007), and, most recently, three species were described from Bhutan (Gittenberger et al. 2017, 2020). Here, we describe a fourth species from Bhutan and present, for the first time, a map of all known records of the genus (see Fig. 1 and Table 1). As usual, it is unknown where snails may have been looked for in vain and thus our distribution maps (Figs 1, 6) may represent human sampling activity rather than the real range of *Erhaia*. Contrary to Gittenberger et al. (2020), in the absence of DNA data, we refer to *E.chandeshwariensis* Nesemann & S. Sharma, 2007 as a species closely related to *E.nainatalensis* Davis & Rao, 1997. The shells cannot be distinguished, but the type localities are over 600 km apart, which makes conspecificity unlikely in *Erhaia*. Remarkable facts are the allopatric distribution and diversification in this genus in general, the syntopic occurrence of *E.jannei* Gittenberger & Stelbrink, 2020 and *E.pelkiae* Gittenberger & Gyeltshen, 2020 in Bhutan (Fig. 6), and that of *E.banepaensis* Nesemann & S. Sharma, 2007 with either *E.chandeshwariensis* or *E.sugurensis* Nesemann, Shah & Tachamo, 2007 in Nepal (Fig. 5).

#### ﻿ Erhaia norbui sp. nov.

Taxon classificationAnimaliaLittorinimorphaAmnicolidae

http://zoobank.org/07BF6064-F69D-44FB-BFBA-34E849962BAF

Figs 2 , 3–5 , 6

##### Material examined.

***Holotype*.** (Fig. 4) Bhutan • District Haa, Uesu, Naychu, ca. 2700 m a.s.l.; 27°22'33"N 89°17'15"E; Sangay Norbu leg. 2020 (NBCB 1239).

***Paratypes*.** (Figs 5–6) 3 shells (NBCB 1240), 2 shells (RMNH.MOL.511432).

##### Diagnosis.

Shell pale greyish, large for the genus (H > 2 mm), with a globular body whorl and a roundish aperture.

##### Description.

Shell obliquely ovoid, with 3½–3¾ regularly convex whorls that are separated by a deep suture; clearly higher than broad; pale greyish with fine irregular growth lines and some blackish-brown periostracal ridges, one of which runs from the apertural columellar border into the umbilicus. Aperture nearly circular in fully grown specimens, with a continuous, free peristome that is thickened, not reflected; with a minute umbilicus. Protoconch encrusted in all specimens; teleoconch without spiral sculpture.

Measurements of shells with thickened apertural border (n = 6): H 2.3–2.6 mm, B 1.6–1.8 mm. Holotype 2.3×1.7 mm.

Shells of *E.jannei*, which are most similar in shape, are yellowish-brown and a little narrower, with the aperture slightly compressed laterally. The other Bhutanese *Erhaia* species known, i.e., *Erhaiapelkiae* and *E.wangchucki* Gittenberger, Sherub & Stelbrink, 2017, are smaller, i.e. H < 2 mm and H < 2.2 mm, respectively; their shells are less pale, of an elongated ovoid shape and with an elliptical aperture in *E.pelkiae*, or conical shape with a piriform aperture in *E.wangchucki*.

##### Ecology

**(Fig. 2).** The species was found in spring water among abundant watercress. The annual temperature of the water is 9–12 °C, with pH of 7–8.5 and 6.5 mg/l oxygen.

##### Molecular data

**Figure 7.**
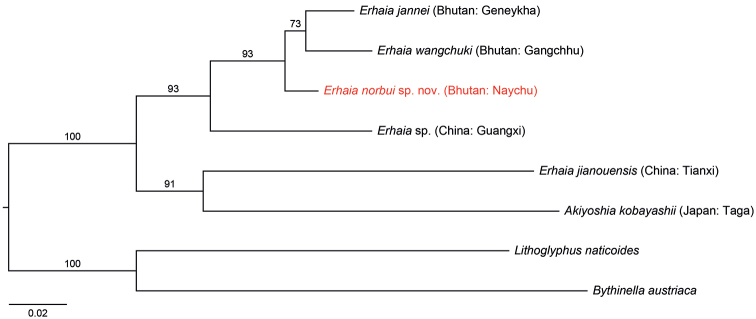
Maximum likelihood tree reconstructed with RAxML BlackBox (Stamatakis et al. 2008; GTR+G substitution model for each partition and 100 bootstrap replicates) based on the COI and 16S rRNA dataset of Liu et al. (2014) and Guan et al. (2008), with new data in red. Numbers on branches denote bootstrap values > 50.

**(Fig. 7).** Both of the individuals (paratypes) that we analyzed genetically shared an identical haplotype for both COI (GenBank acc. no.: OM135616) and 16S rRNA (GenBank acc. no.: OM135244). The uncorrected genetic p-distances between *E.norbui* sp. nov. vs. *E.jannei* and *E.wangchuki* were 3.97% and 5.19%, respectively, for COI, and 1.42% and 1.22%, respectively, for 16S rRNA.

##### Notes.

The three *Erhaia* species from Bhutan form a highly supported clade, with *Erhaia* sp. from China as the sister-group. Interestingly, the species called *E.jianouensis*, from China, and *Akiyoshiakobayashii*, from Japan, form the highly supported sister-group of the remaining *Erhaia* species (see foregoing notes for *Erhaia*). For additional notes regarding the truncatelloidean gastropods of N. India, Nepal, Bhutan, and S. China, in particular the species of *Erhaia*, see also Gittenberger et al. (2020).

##### Etymology.

The epithet *norbui* refers to Mr. Sangay Norbu, who discovered this species.

## Supplementary Material

XML Treatment for Erhaia

XML Treatment for Erhaia norbui
